# Discovery of a selective, state-independent inhibitor of Na_V_1.7 by modification of guanidinium toxins

**DOI:** 10.1038/s41598-020-71135-2

**Published:** 2020-09-09

**Authors:** H. Pajouhesh, J. T. Beckley, A. Delwig, H. S. Hajare, G. Luu, D. Monteleone, X. Zhou, J. Ligutti, S. Amagasu, B. D. Moyer, D. C. Yeomans, J. Du Bois, J. V. Mulcahy

**Affiliations:** 1 SiteOne Therapeutics, South San Francisco, CA 94080 USA; 2 SiteOne Therapeutics, Bozeman, MT 59715 USA; 3 Department of Chemistry, Stanford University, Stanford, CA 94305 USA; 4 Neuroscience Department, Amgen Research, Thousand Oaks, CA 91320 USA; 5 Department of Anesthesiology, Perioperative, and Pain Medicine, Stanford University, Stanford, CA 94305 USA

**Keywords:** Drug discovery, Neuroscience

## Abstract

The voltage-gated sodium channel isoform Na_V_1.7 is highly expressed in dorsal root ganglion neurons and is obligatory for nociceptive signal transmission. Genetic gain-of-function and loss-of-function Na_V_1.7 mutations have been identified in select individuals, and are associated with episodic extreme pain disorders and insensitivity to pain, respectively. These findings implicate Na_V_1.7 as a key pharmacotherapeutic target for the treatment of pain. While several small molecules targeting Na_V_1.7 have been advanced to clinical development, no Na_V_1.7-selective compound has shown convincing efficacy in clinical pain applications. Here we describe the discovery and characterization of ST-2262, a Na_V_1.7 inhibitor that blocks the extracellular vestibule of the channel with an IC_50_ of 72 nM and greater than 200-fold selectivity over off-target sodium channel isoforms, Na_V_1.1–1.6 and Na_V_1.8. In contrast to other Na_V_1.7 inhibitors that preferentially inhibit the inactivated state of the channel, ST-2262 is equipotent in a protocol that favors the resting state of the channel, a protocol that favors the inactivated state, and a high frequency protocol. In a non-human primate study, animals treated with ST-2262 exhibited reduced sensitivity to noxious heat. These findings establish the extracellular vestibule of the sodium channel as a viable receptor site for the design of selective ligands targeting Na_V_1.7.

## Introduction

The voltage-gated sodium ion channel (Na_V_) isoform 1.7 has emerged as a high-interest target for the discovery of non-opioid pain therapeutics based on compelling validation from human genetics and preclinical studies^1^. Na_V_1.7 loss-of-function mutations result in whole-body insensitivity to pain; conversely, gain-of-function variants are associated with episodic extreme pain disorders and small fiber neuropathies^2–5^. Discovery of selective inhibitors of Na_V_1.7 has been challenging due to the structural conservation of off-target Na_V_ isoforms (Na_V_1.1–1.6, Na_V_1.8 and Na_V_1.9), inhibition of which is likely to result in safety liabilities^6–8^.

**Figure 1:**
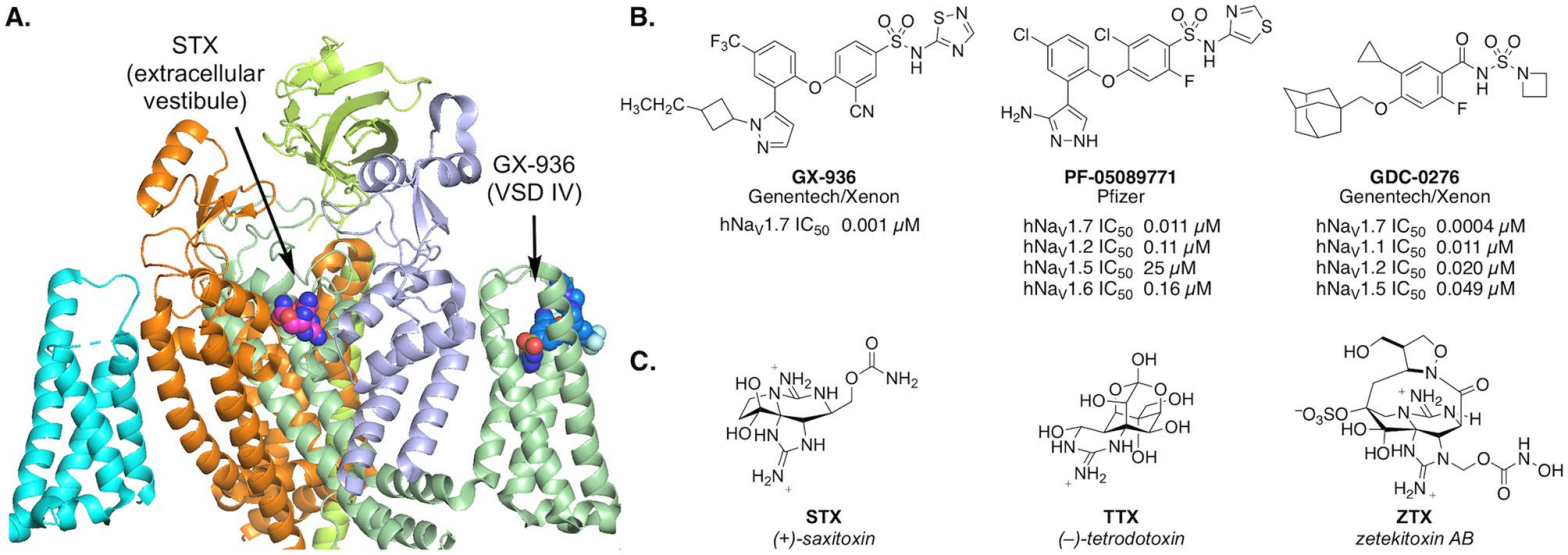
(**A**) Cryo-EM structure of STX bound to human Na_V_1.7-β1-β2 complex (PDB: 6j8g) with GX-936 positioned approximately based on PDB: 5ek0 in Pymol version 2.0.4 (Schrodinger, New York, NY). (**B**) Representative Na_V_1.7 inhibitors that bind VSD IV. (**C**) Natural Na_V_ inhibitors that bind to the extracellular vestibule^18–21^.

Na_V_s are integral membrane proteins expressed in excitable cells that comprise a ~ 260 kD pore-forming α-subunit and up to two accessory β-subunits (Fig. 1A)^9^. The central pore of the α-subunit is encircled by four voltage-sensing domains (VSD I–IV). Channel gating occurs through protein conformational changes in response to membrane depolarization. At least nine discrete binding sites on the Na_V_ α-subunit have been identified for peptides and small molecules that influence ion conductance^10^. The large majority of molecules that engage Na_V_s bind preferentially to a specific conformational state of the channel and show use-dependent activity. Clinical Na_V_ inhibitors (e.g., bupivacaine, lidocaine, carbamazepine) are both state- and frequency-dependent agents that lodge in the intracellular pore of the α-subunit, a site that is highly conserved between isoforms. These drugs rely on local administration to achieve a margin between the desired pharmacodynamic effect and dose-limiting side effects. Certain investigational Na_V_ inhibitors, such as peptide toxins isolated from venomous species, interact with VSDs to alter the kinetics or voltage dependence of channel activation or inactivation^11,12^. Similarly, a class of small molecule aryl and acyl sulfonamide compounds bind to an activated conformation of VSD IV and prevent recovery from inactivation (Fig. 1B)^13–16^. By contrast, cationic guanidinium toxins and peptide cone snail toxins inhibit ion conduction by sterically occluding the extracellular vestibule of the channel pore (Site 1). The former are a unique collection of small molecule natural products exemplified by saxitoxin and tetrodotoxin—high affinity, state-independent blockers against six of nine Na_V_ subtypes (Fig. 1C)^17^.

In the pursuit of isoform-selective inhibitors of Na_V_1.7, two binding sites, the cystine knot toxin site at VSD II and the sulfonamide site at VSD IV, have been heavily interrogated. Certain cystine knot toxins that engage VSD II such as HwTx-IV, Pn3a, and ProTx-II exhibit 6–1,000× selectivity for Na_V_1.7 over other channel isoforms. Potency and selectivity for this target have been improved with synthetic peptide toxin derivatives^22–26^. Among small, Lipinski-type molecules, only the aryl and acyl sulfonamides pioneered by Icagen/Pfizer and subsequently investigated by Amgen, Chromocell, Genentech/Xenon, Merck, and others have shown evidence of significant Na_V_1.7 isoform selectivity^7,16^. Within the sulfonamide series, selectivity levels are > 1,000× over certain off-target isoforms including the cardiac isoform, Na_V_1.5, but generally 10–50× against Na_V_1.2 and Na_V_1.6. Many but not all sulfonamide Na_V_1.7 inhibitors suffer from undesirable pharmaceutical properties, including high plasma protein binding (e.g. > 99.8%), cytochrome p450 inhibition, in vitro hepatotoxicity and high unbound clearance^27,28^, which have hindered clinical development. Although a number of candidates have been advanced to human testing, one compound has been discontinued after a Phase 2 study likely due to limited efficacy (PF-05089771); others have been discontinued after Phase 1 trials for reasons that may be related to safety liabilities such as elevated expression of liver transaminases and hypotension (GDC-0276)^29,30^.

Electrophysiology studies with naturally occurring Site 1 ligands against different wild-type and mutant Na_V_ isoforms have identified the extracellular vestibule of Na_V_1.7 as a promising locus for selective inhibitor design^31–33^. The outer mouth of the channel is formed from residues that link the S5–S6 helices (referred to as pore loops) from each of the four domains. The domain III pore loop of human Na_V_1.7 contains a T1398/I1399 sequence motif that is not present in other human Na_V_ subtypes (which contain MD at equivalent positions, Suppl Table 1)^31^. Comparison of the amino acid sequence of the domain III pore loop across species indicates that the sequence motif in hNa_V_1.7 is unique to primates. The half-maximal inhibitory concentration (IC_50_) value for saxitoxin (STX) is markedly altered (250-fold change) depending on the presence or absence of the T1398 and I1399 residues. Against rNa_V_1.4, the IC_50_ of STX is 2.8 ± 0.1 nM compared to 702 ± 53 nM for hNa_V_1.7^31^. Introduction of the alternative domain III pore loop sequence by mutagenesis restores potency (hNa_V_1.7 T1398M/I1399D IC_50_ = 2.3 ± 0.2 nM). These findings suggest that it may be possible to capitalize on structural differences in the extracellular vestibule between hNa_V_ isoforms to design Na_V_1.7-selective inhibitors.

Recent advances in the de novo synthesis of guanidinium toxin analogues have enabled systemic examination of the structure–activity relationship (SAR) properties that govern hNa_V_1.7 potency and isoform selectivity^34–37^. Prior to 2016, the binding orientation of STX proposed in the literature indicated that the C11 methylene carbon was positioned proximally to domain III pore loop residues^38–40^. SAR and mutant cycle analysis studies posited a revised pose in which the C13 carbamate moiety abuts DIII^32^. This revised binding pose was recently confirmed by cryoelectron microscopy (cryo-EM) structures of STX bound to Na_V_PaS and hNa_V_1.7^18,41^. In the present study, analogues of STX substituted at both the C11 and C13 positions were investigated to understand the requirements for selective inhibition of hNa_V_1.7. These efforts led to the discovery of ST-2262, a potent and selective inhibitor of Na_V_1.7 that reduces sensitivity to noxious heat in a preliminary study in non-human primates (NHPs).

## Results

### Discovery of ST-2262

**Figure 2:**
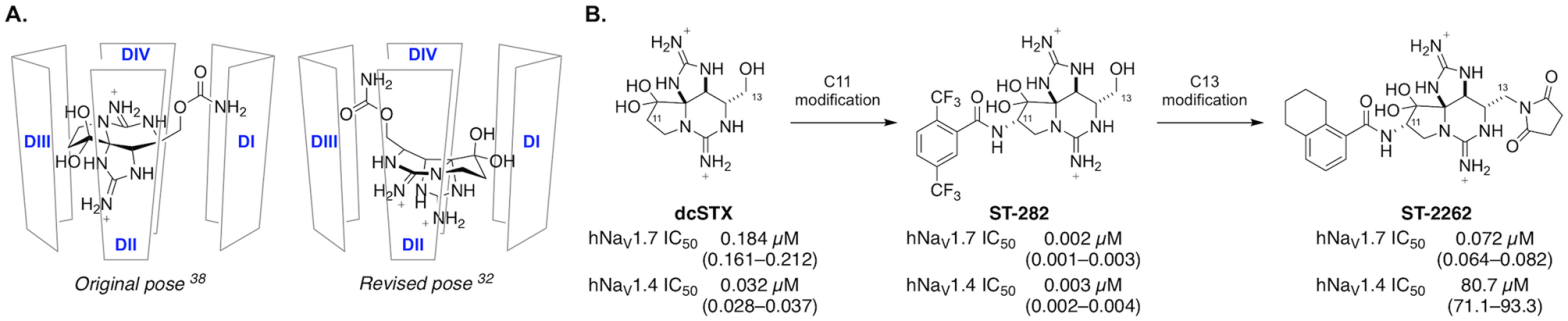
(**A**) The consensus pose for binding of STX in the extracellular vestibule of Na_V_ oriented C11 in proximity to the DIII pore loop prior to 2016^38^. A revised pose based on mutant cycle analysis and recent cryo-EM structures orients the C13 carbamate near DIII^32,41^. (**B**) ST-2262 was discovered by a rational design strategy aimed at identifying functional groups that interact with the DIII T1398/I1399 sequence motif unique to primate Na_V_1.7. Values are mean (95% CI).

ST-2262 was discovered through a rational design strategy aimed at identifying derivatives of natural bis-guanidinium toxins that preferentially inhibit hNa_V_1.7 over other off-target hNa_V_ isoforms^31^. Mutagenesis, homology modeling, and docking studies conducted prior to 2016 suggested that bis-guanidinium toxins orient in the outer mouth of the channel with the C11 methylene center positioned toward the domain III pore loop of Na_V_ (Fig. 2A, Original pose)^38–40^. Exploration of substitution at C11 of decarbamoyl saxitoxin (dcSTX) led to the identification of a series of analogues bearing aryl amide groups at this site. Certain compounds, as exemplified by ST-282, show excellent potency against hNa_V_1.7 but minimal selectivity (~ 1:1) over off-target isoforms such as hNa_V_1.4 (Fig. 2B). The finding that hNa_V_1.7 isoform selectivity could not be achieved by modification of the C11 substituent led us to investigate SAR at alternative positions. These studies followed evidence that the proper binding orientation of STX is rotated ~ 180° from earlier models, thus placing the C13 substituent in close proximity to domain III (Fig. 2A, Revised pose)^32^.

Derivatives of STX bearing amide, carbamate, ester, ether, and urethane substituents at the C13 position were prepared in an effort to identify compounds with improved selectivity for hNa_V_1.7. Insight from studies of a naturally-occurring STX C13 acetate congener, STX-OAc, helped guide selection of compounds for synthesis (Suppl Figure 1)^32^. The difference in potencies between STX and STX-OAc is striking considering that these two structures vary at a single position (NH_2_ → CH_3_). Following this lead, we explored substituents at C13 that could replace the hydrolytically unstable acetate group. Ultimately, the C13 succinimide was discovered as a suitable acetate isostere, which was paired with a C11 tetrahydronaphthyl amide to generate ST-2262, the focus of the present study.

### ST-2262 is a potent and selective inhibitor of hNa_V_1.7

**Figure 3:**
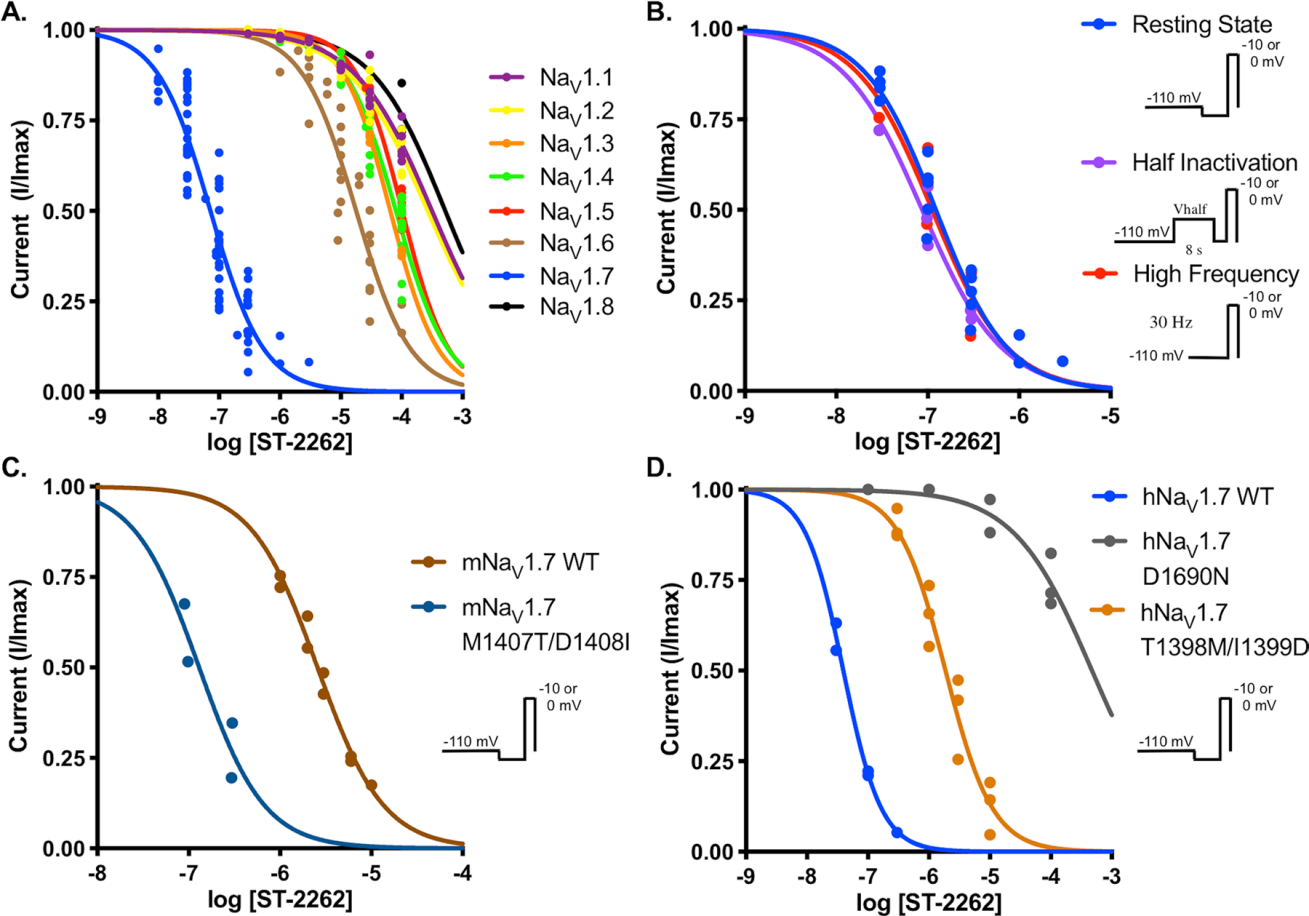
(**A**) Dose–response curves for the inhibitory effect of ST-2262 on Na_V_1.1–Na_V_1.8 stably expressed in CHO or HEK293 cells using a single-pulse (resting state) protocol with a 10 ms pulse from a holding potential of – 110 mV to voltage at peak activation (– 20 to + 10 mV). Na_V_1.X IC_50_ (in µM, mean, 95% CI). Na_V_1.1: > 100; Na_V_1.2: > 100; Na_V_1.3: 65.3, 62.7–68.1; Na_V_1.4: 80.7, 71.1–93.3; Na_V_1.5: > 100; Na_V_1.6: 17.9, 14.8–22.1; Na_V_1.7: 0.072, 0.064–0.082; Na_V_1.8: > 100. (**B**) Comparison of dose–response relationship of ST-2262 inhibition against Na_V_1.7 using different stimulation protocols: resting state; two-pulse protocol contained an 8 s conditioning step to the voltage at half-inactivation, followed by a 20 ms step to voltage at full activation (half-inactivation protocol)^16^; high frequency single-pulse protocol stimulated at 30 Hz. Na_V_1.7 IC_50_ (in µM, mean, 95% CI). Resting state: 0.123, 0.104–0.145; half-inactivation: 0.087, 0.056–0.120; high frequency: 0.112, 0.015–0.357. (**C**) Comparison of dose–response relationship of Na_V_1.7 inhibition against WT mNa_V_1.7 and M1407T/D1408I mNa_V_1.7 on a resting state protocol. mNa_V_1.7 IC_50_ (in µM, 95% CI). WT: 2.57, 2.30–2.87; M1407T/D1408I: 0.130, 0.055–0.307. (**D**) Comparison of dose–response of ST-2262 against transiently expressed hNa_V_1.7 WT, hNa_V_1.7 D1690N, and hNav1.7 T1398M/I1399D. IC_50_ (in µM, mean, 95% CI). WT: 0.039, 0.032–0.047; D1690N: > 100; T1398M/I1399D: 1.87, 1.47–2.39.

The potency of ST-2262 against hNa_V_1.7 stably expressed in HEK293 cells was assessed by manual patch clamp electrophysiology with a voltage-protocol that favors the resting state of the channel. Using a stimulation protocol with a holding potential of – 110 mV and a stimulus frequency of 0.33 Hz, the IC_50_ of ST-2262 against hNa_V_1.7 was measured at 0.072 µM (95% confidence interval (CI) 0.064–0.082) (Fig. 3A, Suppl Table 2). Potencies against off-target sodium channel isoforms (hNa_V_1.1–1.6, hNa_V_1.8) were determined following a similar protocol. Activity against hNa_V_1.9 was not evaluated due to the difficulty of expressing this subtype heterologously^42^. ST-2262 was determined to be > 200-fold selective over hNa_V_1.6 (IC_50_ = 17.9 µM, 95% CI 14.8–22.1), > 900-fold selective over hNa_V_1.3 (IC_50_ = 65.3 µM, CI 62.7–68.1), and > 1,000-fold selective over all other Na_V_ isoforms tested. Similar IC_50_ values against the eight hNa_V_ subtypes were obtained in an independent study using the PatchXpress automated electrophysiology platform (Suppl Table 3).

Exposure of hNa_V_1.1 and hNa_V_1.2 to high concentrations of ST-2262 (10–100 µM) resulted in a reduction of the rate of fast inactivation; a similar effect was noted, albeit to a lesser degree, with hNa_V_1.3 and hNa_V_1.4 (Suppl Figure 2). Lower concentrations of ST-2262 (1–3 µM), which remain sufficiently high to achieve > 90% inhibition of hNa_V_1.7, had no measurable effect on fast inactivation of hNa_V_1.1 and hNa_V_1.2. It is possible that elevated concentrations of ST-2262 result in a secondary mode of binding against these Na_V_ subtypes, however, efforts have not been made to examine such a mechanism at this time. To our knowledge, changes in the rate of fast inactivation have not been observed with STX.

To investigate whether the potency of ST-2262 was dependent on the membrane holding potential or frequency of stimulus, an IC_50_ value was measured against hNa_V_1.7 using a two-pulse protocol with a pre-pulse to the voltage at half-inactivation (8 s step) and with a protocol that depolarizes the cell at high frequency (30 Hz stimulus). The potency of ST-2262 was not appreciably altered using either stimulation protocol (IC_50_ = 0.087 µM, 0.056–0.120 and IC_50_ = 0.112 µM, 0.015–0.357, respectively; Fig. 3B, Suppl Table 4). These results indicate that ST-2262 is a selective, use-independent inhibitor of hNa_V_1.7.

### Species variation in potency and mutagenesis

The potency of ST-2262 was assessed against a panel of species variants of Na_V_1.7, including mouse, rat, and cynomolgus monkey (Suppl Table 5). Consistent with the hypothesis that Na_V_1.7 potency is affected by the presence of the T1398/I1399 sequence motif in the DIII pore loop, the IC_50_ of ST-2262 against cynoNa_V_1.7 (0.101 µM, 0.073–0.140) was similar to human. In contrast, ST-2262 was > 50 × less potent against mouse (IC_50_ = 3.78 µM, 3.23–4.43) and rat Na_V_1.7 (IC_50_ = 4.95 µM, 4.17–5.87) than the human ortholog. Affinity was restored within twofold of the hNa_V_1.7 potency by introduction of domain III MD-TI mutations to mouse Na_V_1.7 (IC_50_ = 0.130 µM, 0.055–0.307; Fig. 3C, Suppl Table 6).

Multiple lines of evidence suggest that ST-2262 binds to the extracellular vestibule of the sodium channel (i.e., Site 1) including: (i) the structural similarity of ST-2262 to natural bis-guanidinium toxin ligands, (ii) the state- and frequency-independent mode of Na_V_ inhibition that is characteristic of extracellular pore blockers, and (iii) the influence of DIII pore loop residues on potency. To gain additional support that ST-2262 binds to the outer pore of Na_V_, we generated a point mutant of hNa_V_1.7, D1690N, at a position known to significantly destabilize binding of STX^39^. The domain IV residue D1690 forms a critical bridged hydrogen bond with the C12 hydrated ketone of STX^39,41^. We also measured potency against the hNa_V_1.7 T1398M/I1399D double mutant to directly confirm that the domain III TI sequence motif contributes to hNa_V_1.7 affinity^31^. The introduction of other point mutations to Na_V_1.7 was attempted (Y362S and E916A), but these variants proved challenging to express^39,43^. ST-2262 exhibited a > 1,000-fold loss in potency against hNa_V_1.7 D1690N (IC_50_ > 100 µM) and a ~ 48-fold loss against hNa_V_1.7 T1398M/I1399D (IC_50_ = 1.87 µM, 1.47–2.39) compared to the wild-type channel (IC_50_ = 0.039 µM, 0.032–0.047; Fig. 3D, Suppl Table 6). Collectively, these results indicate that ST-2262 binds to the extracellular vestibule of Na_V_1.7, displaying significant species variation in potency and isoform selectivity in large part due to molecular interactions with residues T1398 and I1399, which are unique to human and non-human primate Na_V_1.7 orthologs^31,32^.

### ST-2262 increases withdrawal latency in a nonhuman primate model of thermal pain

Mice and humans with genetic Na_V_1.7 loss-of-function are profoundly insensitive to noxious heat^2,3,44–46^. To understand whether pharmacological block of Na_V_1.7 affects noxious thermal sensitivity, we conducted an initial evaluation (n = 4) of the effect of ST-2262 in a non-human primate (NHP) model of acute thermal pain. Experiments were approved by the Montana State University institutional animal care and use committee and performed in accordance with institutional, national, and international guidelines and regulations. It is not possible to study the influence of ST-2262 on acute thermal pain in rodents as this compound is > 50-fold less potent against Na_V_1.7 in species that lack the T1398/I1399 sequence motif (Suppl Table 5). A NHP model of acute thermal pain was identified that uses a heat lamp to deliver a stimulus to the dorsal surface of the hand of lightly anesthetized cynomolgus macaques and measures the time to withdrawal^47^. Prior to advancing ST-2262 into the NHP acute thermal pain model, a standard battery of preclinical assays was completed to evaluate ADME and pharmacokinetic properties of this compound in cynomolgus macaques (Suppl Table 7). Off-target activity of ST-2262 using a commercially available radioligand binding assay panel against 68 different targets was also measured (LeadProfilingScreen, Eurofins, Taipei, Taiwan). No hits were identified on the off-target panel, defined as > 50% inhibition with 10 µM ST-2262 (Suppl Table 8).

**Figure 4:**
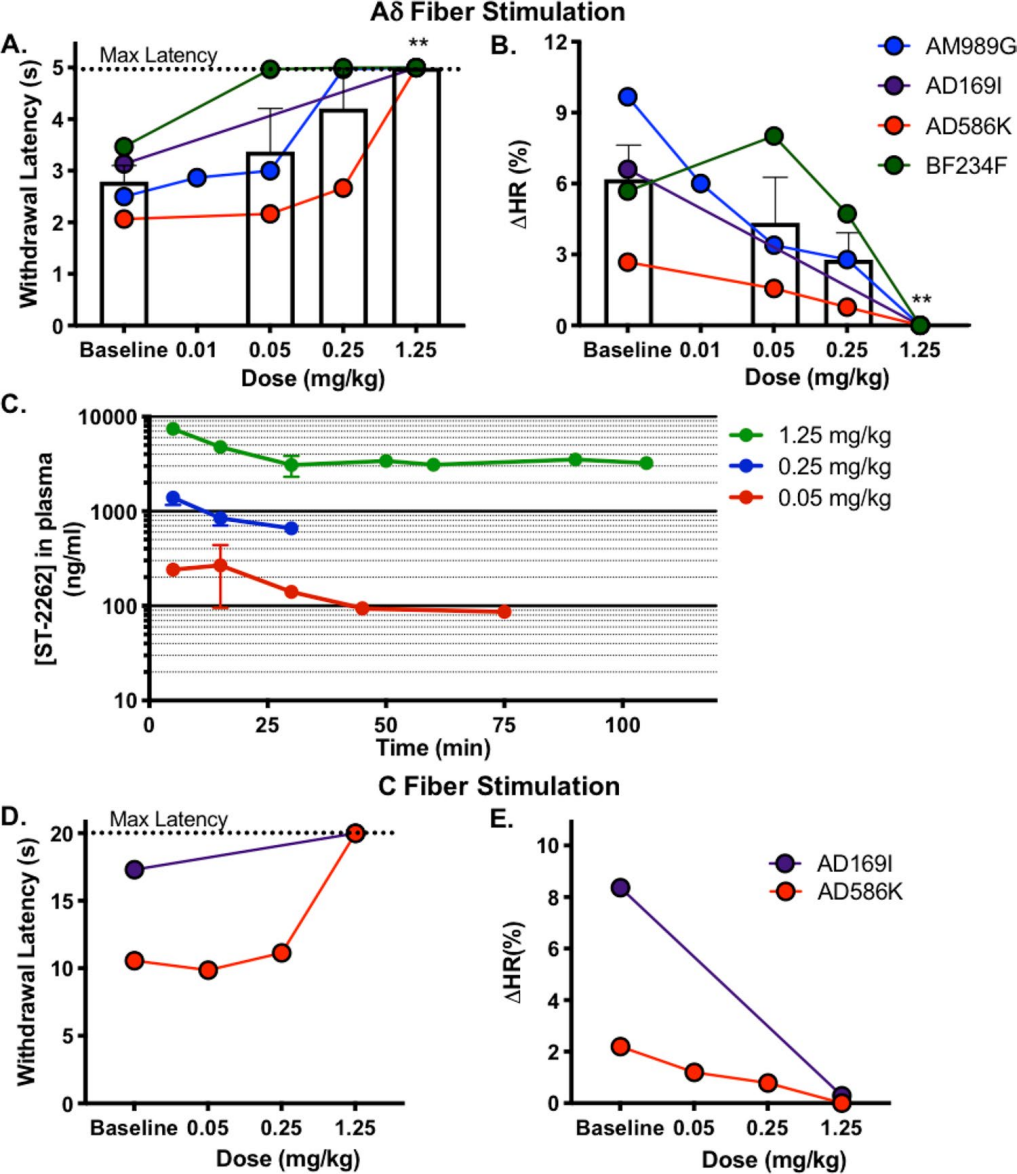
ST-2262 increases withdrawal latency and reduces thermal evoked heart rate increase in a non-human primate noxious heat model. (**A**,**B**) Individual subject data points showing changes in withdrawal latency (**A**) and transient change in heart rate (ΔHR) (**B**) following thermal stimuli. Bar graphs are expressed as mean ± SEM. **Dunnett’s multiple comparison test, compared to baseline, p < 0.01. (**C**) Plasma level concentration of ST-2262 in plasma at different doses. (**D**,**E**) A lower heating rate thermal stimulus was presented for a maximum of 20 s, which selectively activates C fibers^47^. In two subjects, the C-fiber-induced hand withdrawal response was replicable for testing. The efficacy endpoints measured were withdrawal latency (**A**) and heart rate change (**B**).

Male cynomolgus monkeys were anesthetized with propofol to a level in which the withdrawal reflex of the hand occurred at a consistent latency of approximately 3 s, a response time that was comparable to the detection of sharp pain from Aδ fibers when tested in prior studies on human volunteers^48,49^. The dorsal surface of the hand was exposed to a thermal stimulus that selectively activates Aδ-fiber nociceptors (Fig. 4A–C)^47,50^. The thermal stimulus was turned off at 5 s to prevent tissue damage. Heart rate was monitored throughout the study, and presentation of the noxious thermal stimuli consistently led to a transient increase in heart rate that peaked seconds after the stimulus and then returned to baseline (ΔHR). Acute noxious thermal stimuli transiently increase heart rate in human subjects; the percent change in heart rate correlates with subjective pain score^51^.

ST-2262 hydrochloride administered IV increased the withdrawal latency to noxious thermal stimuli (Fig. 4A). Efficacy was assessed in one subject at four dose levels (0.01, 0.05, 0.25, 1.25 mg/kg), in two subjects at the three higher dose levels (0.05, 0.25, 1.25 mg/kg), and in one additional subject at the highest dose level only (1.25 mg/kg). At the highest dose of 1.25 mg/kg, all four animals showed no hand withdrawal prior to the 5 s cut-off latency (Fig. 4A), a significant increase in withdrawal latency compared to baseline values (Mixed effects model: F(3,7) = 7.468, p < 0.05; 0.01 mg/kg was not included in this analysis because only one subject received this dose). The 1.25 mg/kg dose of ST-2262 also almost completely reduced ΔHR (Fig. 4B; Mixed effects model: F(3,7) = 6.654, p < 0.05.)

Plasma samples were obtained from animals to assess the PK/PD relationship between drug exposure and thermal withdrawal latency. We found that 0.25 mg/kg ST-2262 resulted in ~ 1,400 ng/ml in plasma at the 5 min time point (n = 2), which corresponds to 7× the IC_50_ value of ST-2262 against cynoNa_V_1.7, corrected for plasma protein binding (cyno PPB = 73.5%). The unbound exposure of drug was reduced to 3.4 × cynoNa_V_1.7 IC_50_ at the 30 min time point. At a dose of 1.25 mg/kg, the total plasma concentration was ~ 7,000 ng/ml at 5 min (n = 2), which corresponds to an unbound exposure of 32 × cynoNa_V_1.7 IC_50_, and was maintained above 15× cynoNa_V_1.7 IC_50_ for over 100 min (Fig. 4C). Lumbar CSF samples collected from two animals receiving the 1.25 mg/kg dose indicated that ST-2262 was peripherally restricted, with CSF:plasma ratios < 10^–3^ (n = 2; [ST-2262] 0.8, < 0.5 ng/ml in CSF).

By adjusting radiant heat parameters, the noxious heat model can be used to selectively assess responses to cutaneous C-fiber nociceptor activation, which produces a burning pain in volunteers^48,49^. The effect of ST-2262 on C-fiber induced hand withdrawal and heart rate change was investigated on two cynomolgus subjects^47^. As with the Aδ nociceptive response, 1.25 mg/kg ST-2262 completely abolished the C-fiber-mediated hand withdrawal and ΔHR (Fig. 4D,E). Collectively, these results are consistent with the hypothesis that pharmacological block of Na_V_1.7 reduces sensitivity to noxious heat, phenotypically analogous to studies of Na_V_1.7 loss-of-function in CIP patients^2^. In addition, analysis of the PK/PD relationship of ST-2262 in this model provides insight into the level of Na_V_1.7 target occupancy that may be necessary to achieve a pharmacodynamic effect. Recognizing the limited number of animals tested due to the challenge of working with non-human primates, additional work is warranted to further define the relationship between pharmacological inhibition of Na_V_1.7 and sensitivity to noxious thermal stimuli.

## Discussion

The finding that humans lacking functional Na_V_1.7 exhibit an inability to experience pain raises the intriguing possibility that selective inhibitors of Na_V_1.7 may be potent analgesics^1–3^. In the present study, we describe the discovery and characterization of ST-2262, a selective pore blocker of hNa_V_1.7 advanced through rational modification of a natural small molecule toxin lead, STX. In whole cell voltage clamp recordings, ST-2262 exhibited > 200-fold selectivity for hNa_V_1.7 over hNa_V_1.1–1.6 and hNa_V_1.8. The selectivity of ST-2262 was not examined against hNa_V_1.9, a channel subtype that is difficult to express in heterologously. hNa_V_1.9 contains a residue in the domain I p-loop, S360, that confers resistance to STX and lacks the domain III threonine/isoleucine sequence motif that is essential for high potency of ST-2262 against hNa_V_1.7. Thus, inhibition of Na_V_1.9 by ST-2262 is unlikely^42^.

The properties of ST-2262 are in contrast to other preclinical and clinical inhibitors of Na_V_1.7, which preferentially bind to an inactivated conformation(s) of the channel^52^. Mutagenesis experiments indicate that specific residues in the extracellular pore of Na_V_1.7, including a two amino acid sequence variation in the domain III pore loop that is unique to primates, are required for ST-2262 binding to cyno- and human Na_V_1.7^31,39^. These findings establish the extracellular vestibule of Na_V_1.7 as a viable receptor site for the design of potent and selective channel inhibitors.

Whereas congenital insensitivity to pain in humans is the result of complete and permanent Na_V_1.7 loss-of-function, inhibition by small molecule agents is incomplete and transient. This difference raises several important questions regarding the pharmacology of Na_V_1.7: (1) is transient inhibition sufficient for analgesia, (2) what level of target engagement is required for efficacy, and (3) what anatomic compartment(s) must be accessed? In light of the preliminary nature of the behavioral studies conducted with ST-2262, the present study does not yield definitive answers to these questions. Nevertheless, the finding that NHPs administered ST-2262 exhibited reduced sensitivity to noxious thermal stimuli is consistent with the view that transient inhibition of Na_V_1.7 is sufficient to produce analgesia^45^. Furthermore, recognizing that ST-2262 is a polar small molecule with low membrane permeability and therefore unlikely to reach efficacious concentrations in the CNS (analysis of CSF samples obtained during NHP experiments gave a CSF:plasma ratio of < 10^–3^), the observed effects on thermal withdrawal latency and ΔHR are likely the result of peripheral inhibition. Our findings, however, do not rule out an additional role for Na_V_1.7 at the central terminals of primary afferents or in dorsal horn neurons, as has been suggested^53^.

In the present study, the effect of ST-2262 on withdrawal latency to noxious heat was measured in NHPs at doses of 0.01, 0.05, 0.25 and 1.25 mg/kg IV. Doses of 0.05, 0.25 and 1.25 mg/kg resulted in unbound plasma concentrations of ST-2262 of 0.7×, 3.4× and 16× the IC_50_ value against cynoNa_V_1.7 at a time point 30 min following drug administration. Assuming a unitary Hill coefficient, which is consistent with the dose–response relationship for ST-2262 in whole cell recordings against cyno- and human Na_V_1.7, these unbound exposures correspond to 41%, 78% and 94% inhibition of Na_V_1.7, respectively. Further work to understand whether a similar relationship exists between Na_V_1.7 target occupancy and analgesic pharmacodynamic effects in other preclinical pain models is ongoing.

## Conclusion

Na_V_1.7 remains a compelling target for the development of non-opioid analgesics based on evidence from human genetics and rodent knock-out studies^2,3,44,45^. A major challenge in the pursuit of safe and effective Na_V_1.7 inhibitors has been the identification of small molecules that are selective over off-target proteins, including other Na_V_ isoforms, to achieve a suitable margin of safety. Prior efforts to develop high precision Na_V_1.7 inhibitors have largely focused on a class of aryl and acyl sulfonamides that bind preferentially to VSD IV and impede recovery from inactivation^7^. In the present study, we disclose ST-2262, a synthetic analogue of natural bis-guanidinium toxins that lodges in the extracellular vestibule of the channel (Site 1) and occludes ion passage. A preliminary PK/PD study involving intravenous administration of ST-2262 to four cynomolgus subjects demonstrated increased withdrawal latency to noxious heat. Collectively, our findings validate the extracellular mouth of the sodium channel as a tractable receptor site for selective ligand design and provide insight into the distribution and target occupancy requirements for drug efficacy mediated by inhibition of Na_V_1.7.

## Supplementary information

Supplementary Information.

## Data Availability

Additional raw data are available from the corresponding author on reasonable request.
